# Data modelling of subsistence retail consumer purchase behavior in South Africa

**DOI:** 10.1016/j.dib.2022.108094

**Published:** 2022-03-26

**Authors:** Valencia Melissa Zulu, Andriaan Mpho Nkuna

**Affiliations:** School of Business Sciences, Marketing Department, University of the Witwatersrand, Johannesburg, South Africa

**Keywords:** Bottom of the pyramid, Subsistence consumer, Retailing, Purchase behavior, South African Townships

## Abstract

The purpose of the data is to model the purchase behavior of the subsistence consumer within the retail environment in one of the largest townships in South Africa. The data was collected using a self-administered questionnaire from a sample of 281 consumers. The Partial Least Squares Structural Equation Modelling (PLS-SEM) approach was adopted using the SmartPLS 3 software to analyze the data. The insights from the dataset identify convenience, price sensitivity, perceived product quality, customer trust, and perceived value as factors that stimulate purchase behavior. Furthermore, perceived value only mediates the relationship between perceived product quality and purchase intention. Researchers could use the data to position customer trust as a dependent variable to unearth more valuable insights. Additionally, the segment in question is also known to be price-sensitive. It would be intriguing to find out the role of price sensitivity as a moderator.

## Specifications Table

**Table:** 

Subject	Marketing
Specific subject area	Bottom of the pyramid, consumer behavior, retail environment
Type of data	Tables and figures
How data were acquired	A structured questionnaire was used to collect data from consumers in one of the largest townships in South Africa
Data format	Raw, descriptive, and analyzed
Parameters for data collection	The sample consisted of grocery store consumers from one of the largest townships in South Africa, Soweto, in Johannesburg. Soweto is an acronym for South-Western Townships and comprises several periurban townships
Description of data collection	Face-to-face self-administered questionnaires were distributed to participants in different settings. This included outside and inside the grocery stores and in the comfort of their homes. The purpose of the research was explained to participants, and consent was obtained before distributing the survey. A non-probability convenience sampling technique was used as there was no database to draw from for a probability sampling approach to be possible. The research data and questionnaire are available in the repository [8]. The questionnaire consists of Section A (demographic information), and Section B (measurement instruments). The data is quantitative and includes descriptive statistics and responses based on a 5-point Likert scale.
Data source location	University of the Witwatersrand, Johannesburg, South Africa
Data accessibility	Repository name: Mendeley Data Data identification number: 10.17632/5z37z85jck.1 Direct URL to data: https://data.mendeley.com/datasets/5z37z85jck/1

## Value of the Data

• The dataset is essential because it provides insights into the consumer buying behavior of a segment worth billions in terms of spending power. This segment is often called the base of the pyramid, the resource-constrained, the impoverished, and the subsistence consumer. The dataset can be used to identify the consumer behavior factors that influence the purchase of products in retail stores.
• The dataset can benefit researchers in retailing and consumer services as the data provides insights on direct relationships between constructs explored and the mediating effect. The data sheds some light on empathy, convenience, price sensitivity, physical environment, perceived product quality, customer trust, and their influence on perceived value and purchase intention. Furthermore, the data highlights the importance of perceived value as a mediator.
• The dataset is also beneficial to retailers interested in servicing the subsistence consumer because it provides insights into which factors the consumers consider as a stimulator of purchase intention. Secondly, given that the study was carried out in a township setting, small informal retailers can benefit from these insights, which means that small business government agencies and policymakers could use the data to inform strategies to assist the commercialization efforts of the township economy. Such methods will also benefit the rural economies.
• For further insights, the data can be used to identify other constructs that can moderate the relationship between perceived value and purchase intention. For example, the moderating role of price sensitivity, given how price-sensitive the segment is. Customer trust can also take a function of a dependent variable to unearth valuable insights.

## 1 Data Description

**Fig. 1:**
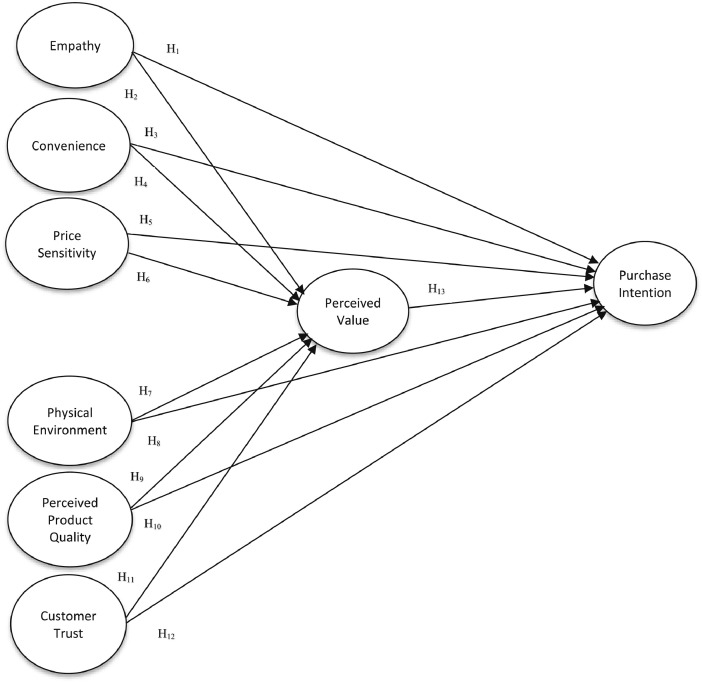
Conceptual Model.

Fig. 1 demonstrates the proposed conceptual model. The conceptual model suggests a connection between empathy, convenience, price sensitivity, physical environment, perceived product quality, customer trust, and purchase intention, and that perceived value mediates these relationships. A self-administered questionnaire (using a 5-Likert scale) was distributed to consumers regarding their purchase behavior. Table 1 provides the demographic profile and characteristics of respondents. Although most respondents were females (47.7%), the gap was insignificant to male respondents (47.3%). The majority of the participants were single (58.4%). There was a fair representation of age distribution as shown in Table 1.

**Table 1:** Profile of respondents.

Category	Characteristic	Frequency	Percentage (%)
Gender	Female	134	47.7
	Male	133	47.3
	I prefer not to say	14	5
Marital Status	Married	79	28.1
	Single	164	58.4
	I prefer not to say	38	13.5
Age	18–22	64	22.8
	23–28	68	24.2
	29–35	47	16.7
	35–49	54	19.2
	50–65	48	17.1
Level of education	No formal education	34	12.1
	Basic education	128	45.6
	Diploma	69	24.6
	Degree	47	16.7
	Postgraduate degree	3	1.1
Employment status	Employed	147	52.3
	Unemployed	134	47.7
Type of customer	Regular	232	82.6
	Need-based	49	17.4
Shopping frequency	1–2 times per week	112	39.9
	2–3 times per week	75	26.7
	3–4 times per week	46	16.4
	5–6 times per week	26	9.3
	6–7 times per week	22	7.8

**Fig. 2:**
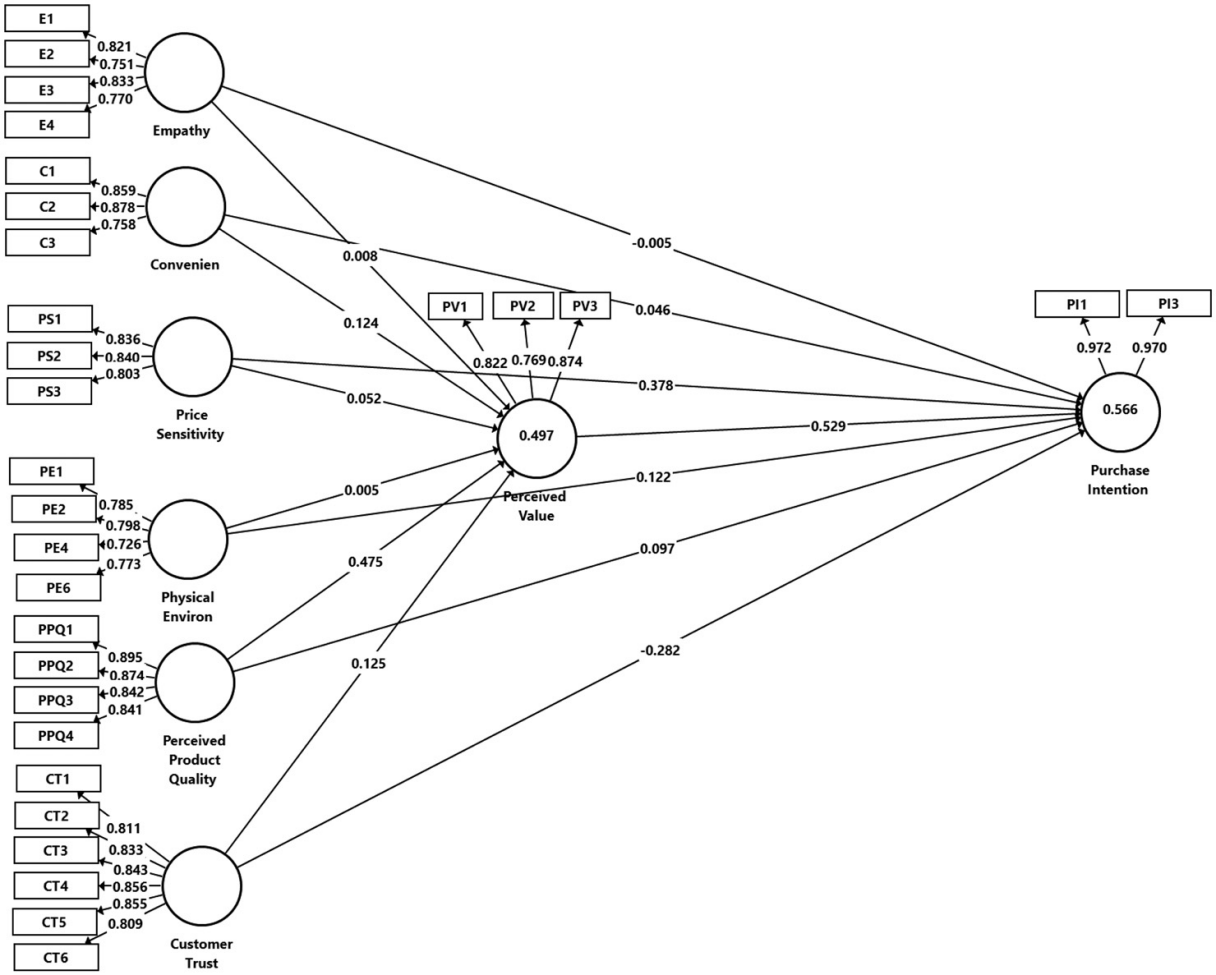
Measurement model output

**Fig. 3:**
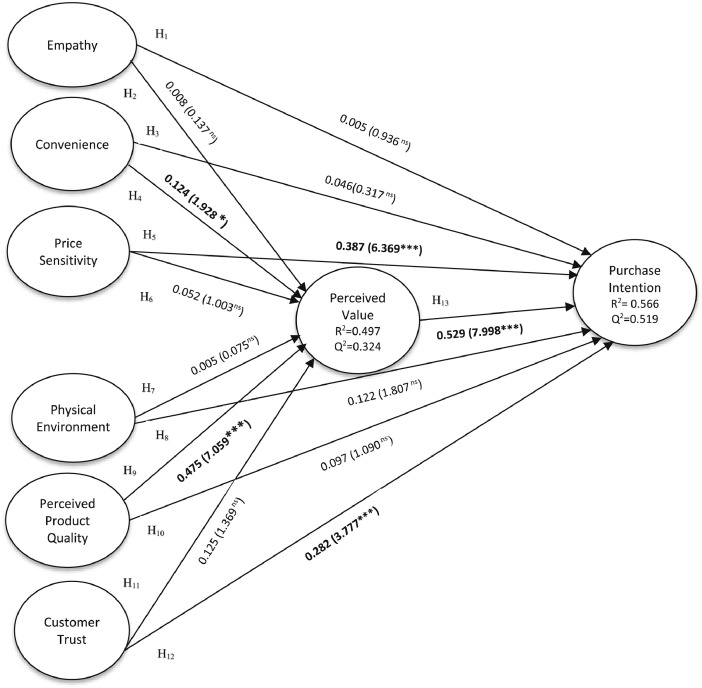
Structural model output.

On the other hand, the majority of the respondents had basic education. Regarding employment status, 52.3% were employed, and 47.7% were unemployed. About 82.6% of consumers indicated that they were regular customers of the grocery stores, with approximately 40% of the consumers indicating that they visit the grocery stores at least 1–2 times per week, and 27% visit the store 2–3 times per week. Table 2 outlines the measurement instruments which were adapted from prior studies. Furthermore, Table 3 shows the assessment of the reflective model, which includes construct reliability and validity tests, while Fig. 3 presents the output of the measurement models with relevant statistics. Tables 4, 5, and 6 show the data analysis for the discriminant validity test, and Fig. 3 demonstrates the structural model output, highlighting the R^2^ and Q^2^ values. Additionally, Table 7 provides a detailed insight into the hypotheses testing in terms of the direct relationships, and Table 8 provides the mediation assessment and indirect effects. The questionnaire and data are provided on Mendely Data. Fig. 2 demonstrates the measurement model output (indicator loadings).

**Table 2:** Measurement instruments.

Construct		Adapted Items	Source
Empathy	E1	The employees of the grocery store understand the specific needs of their customers	[1]
	E2	The grocery store understands what I need and strives to accommodate me	
	E3	The grocery store has employees who give customers personal service	
	E4	The employees of the grocery stores are very efficient	

Convenience	C1	The grocery store layout makes it easy for me to find what I need	[2]
	C2	The grocery store layout makes it easy for me to move around	
	C3	The grocery store always has merchandise available	

Price Sensitivity	PS1	I will continue to buy from the grocery store even if prices increase	[3]
	PS2	I am willing to pay a higher price for the benefit of having the grocery store located close to me	
	PS3	I am willing to stick with the grocery store and not travel to other competitors outside the township who might offer reasonable prices	

Physical Environment	PE1	The store overall has an appealing looking appearance	[4]
	PE2	The grocery store provides a clean shopping environment	
	PE3	The grocery store has wide and open aisles	
	PE4	The grocery store has well-marked aisle signage	
	PE5	The grocery store provides a pleasant shopping environment	
	PE6	The grocery store's environment feels safe and secure	
Perceived Product Quality	PPQ1	The overall quality of products I buy from the grocery store is good	[4]
	PPQ2	The quality of the produce department in the grocery store is good	
	PPQ3	The quality of the meat department in the grocery store is good	
	PPQ4	The quality of in-store bakery is good	

Customer Trust	CT1	The grocery store always meets my expectations	[5]
	CT 2	I can count on the store to meet my grocery needs	
	CT 3	The grocery store is reliable	
	CT4	The grocery store can always be trusted	
	CT5	The grocery store consistently provides good quality products and services	
	CT6	The grocery store's offerings are worth the money I spend	
	CT7	The grocery store helps me save time	

Perceived Value	PV1	The grocery store products have a good value for money	[6]
	PV2	The grocery store products are affordable	
	PV3	In this grocery store, compared to other stores outside the township, I can save money	

Purchase Intention	PI1	I intend to purchase from this grocery store	[7]
	PI2	I would like to repeat my experience in this kind of grocery store	
	PI3	I would purchase from this grocery store in the future	
	PI4	I would recommend purchasing in this grocery store to others

**Table 3:** Reflective measurement model analysis.

Constructs		Outer Loadings (> 0.7)	Cronbach's Alpha (> 0.7)	rho_A (> 0.7)	Composite Reliability (CR) (> 0.7)	Average Variance Extracted (AVE) (> 0.5)	Variance Inflation Factor (VIF) (< 5)
Empathy (E)	E1	0.821	0.808	0.822	0.872	0.631	1.924
	E2	0.751					1.799
	E3	0.833					1.852
	E4	0.770					1.648

Convenience (C)	C1	0.859	0.780	0.797	0.872	0.695	1.760
	C2	0.878					1.879
	C3	0.758					1.432

Price Sensitivity (PS)	PP2	0.836	0.769	0.771	0.866	0.683	1.750
	PP3	0.840					1.862
	PP4	0.803					1.376

Physical Environment (PE)	PE1	0.785	0.775	0.790	0.854	0.595	1.594
	PE2	0.798					1.463
	PE4	0.726					1.498
	PE6	0.773					1.575

Perceived Product Quality (PPQ)	PPQ1	0.895	0.886	0.893	0.921	0.745	2.631
	PPQ2	0.874					2.517
	PPQ3	0.842					2.214
	PPQ4	0.841					2.055

Customer Trust (CT)	CT1	0.811	0.913	0.916	0.932	0.697	2.317
	CT2	0.833					2.611
	CT3	0.843					2.501
	CT4	0.856					3.037
	CT5	0.855					2.599
	CT6	0.809					2.167

Perceived Value (PV)	PV1	0.822	0.761	0.767	0.863	0.677	1.613
	PVE	0.769					1.404
	PV3	0.874					1.836

Purchase Intention (PI)	PI1	0.972	0.940	0.941	0.971	0.943	4.660
	PI3	0.970					4.660

**Table 4:** Discriminant validity - Fornell-Larcker criterion.

	C	CT	E	PV	PPQ	PE	PS	PI
C	0.834							
CT	0.637	0.835						
E	0.523	0.571	0.794					
PV	0.558	0.602	0.405	0.823				
PPQ	0.690	0.762	0.494	0.688	0.863			
PE	0.668	0.752	0.583	0.575	0.770	0.771		
PS	0.357	0.517	0.441	0.381	0.450	0.426	0.826	
PI	0.442	0.423	0.358	0.664	0.539	0.478	0.543	0.971

Note(s): Convenience (C); Customer Trust (CT); Empathy (E); Perceived Value (PV); Perceived Product Quality (PPQ); Physical Environment (PE); Price Sensitivity (PS); Purchase Intention (PI).

**Table 5:** Discriminant validity - Heterotrait-monotrait ratio (HTMT).

	C	CT	E	PV	PPQ	PE	PS	PI
C								
CT	0.762							
E	0.645	0.664						
PV	0.723	0.722	0.486					
PPQ	0.830	0.849	0.565	0.832				
PE	0.869	0.888	0.735	0.729	0.916			
PS	0.449	0.609	0.573	0.491	0.543	0.543		
PI	0.508	0.453	0.406	0.784	0.586	0.551	0.631	

Note(s): Convenience (C); Customer Trust (CT); Empathy (E); Perceived Value (PV); Perceived Product Quality (PPQ); Physical Environment (PE); Price Sensitivity (PS); Purchase Intention (PI).

**Table 6:** Cross-loadings.

	C	CT	E	PE	PI	PPQ	PS	PV
C1	**0.859**	0.516	0.429	0.533	0.368	0.615	0.302	0.514
C2	**0.878**	0.513	0.465	0.564	0.456	0.605	0.319	0.456
C3	**0.758**	0.583	0.415	0.590	0.262	0.497	0.268	0.422
CT1	0.509	**0.811**	0.521	0.647	0.243	0.599	0.397	0.445
CT2	0.511	**0.833**	0.461	0.591	0.388	0.621	0.371	0.466
CT3	0.521	**0.843**	0.506	0.611	0.293	0.624	0.455	0.579
CT4	0.449	**0.856**	0.416	0.585	0.357	0.609	0.403	0.501
CT5	0.621	**0.855**	0.492	0.718	0.410	0.666	0.434	0.538
CT6	0.569	**0.809**	0.469	0.611	0.408	0.692	0.521	0.469
E1	0.465	0.496	**0.821**	0.486	0.255	0.448	0.285	0.403
E2	0.307	0.425	**0.751**	0.383	0.237	0.255	0.442	0.159
E3	0.407	0.398	**0.833**	0.510	0.317	0.422	0.303	0.345
E4	0.448	0.493	**0.770**	0.451	0.317	0.398	0.420	0.319
PE1	0.508	0.543	0.458	**0.785**	0.318	0.536	0.270	0.439
PE2	0.534	0.635	0.395	**0.798**	0.429	0.683	0.370	0.556
PE4	0.530	0.569	0.455	**0.726**	0.330	0.513	0.347	0.314
PE6	0.495	0.563	0.514	**0.773**	0.379	0.613	0.325	0.418
PI1	0.429	0.400	0.361	0.476	**0.972**	0.538	0.521	0.662
PI3	0.430	0.423	0.335	0.451	**0.970**	0.508	0.535	0.627
PPQ1	0.593	0.643	0.451	0.666	0.543	**0.895**	0.488	0.657
PPQ2	0.576	0.745	0.377	0.682	0.419	**0.874**	0.347	0.578
PPQ3	0.651	0.661	0.474	0.647	0.448	**0.842**	0.375	0.507
PPQ4	0.573	0.595	0.407	0.668	0.439	**0.841**	0.330	0.618
PS1	0.331	0.423	0.438	0.433	0.445	0.440	**0.836**	0.327
PS2	0.191	0.380	0.306	0.274	0.379	0.390	**0.840**	0.299
PS3	0.344	0.467	0.343	0.340	0.508	0.293	**0.803**	0.314
PV1	0.461	0.567	0.433	0.535	0.496	0.604	0.384	**0.822**
PV2	0.415	0.490	0.186	0.436	0.522	0.511	0.199	**0.769**
PV3	0.498	0.435	0.370	0.451	0.617	0.581	0.347	**0.874**

**Table 7:** Assessment of the structural model.

Hypotheses	Path	Path Coefficient (β)	T-values	P-values	Decision
H_1_	Empathy -> Purchase Intention	−0.005	0.080	0.936 ^ns^	Not supported
H_2_	Empathy -> Perceived Value	0.008	0.137	0.891 ^ns^	Not supported
H_3_	Convenience -> Purchase Intention	0.046	0.317	0.751 ^ns^	Not supported
H_4_	**Convenience -> Perceived Value**	**0.124**	**1.928**	0.054*	**Supported**
H_5_	**Price Sensitivity -> Purchase Intention**	**0.378**	**6.369**	0.000***	**Supported**
H_6_	Price Sensitivity -> Perceived Value	0.052	1.003	0.316 ^ns^	Not supported
H_7_	Physical Environment -> Perceived Value	0.005	0.075	0.940 ^ns^	Not supported
H_8_	Physical Environment -> Purchase Intention	0.122	1.807	0.072 ^ns^	Not Supported
H_9_	**Perceived Product Quality -> Perceived Value**	**0.475**	**7.059**	0.000***	**Supported**
H_10_	Perceived Product Quality -> Purchase Intention	0.097	1.090	0.276 ^ns^	Not supported
H_11_	Customer Trust -> Perceived Value	0.125	1.369	0.171 ^ns^	Not supported
H_12_	**Customer Trust -> Purchase Intention**	**0.282**	**3.777**	0.000***	**Supported**
H_13_	**Perceived Value -> Purchase Intention**	**0.529**	**7.998**	0.000***	**Supported**

Notes: ***p < 0.001; *p < 0.05, (^ns^): not significant.

**Table 8:** Mediation Assessment.

Hypotheses	Path	Path Coefficient (β)	T-values	P-values	Decision
H_14_	Empathy -> Perceived Value-> Purchase Intention	0.004	0.134	0.894 ^ns^	Not supported
H_15_	Convenience -> Perceived Value -> Purchase Intention	0.066	1.836	0.066 ^ns^	Not supported
H_16_	Price Sensitivity -> Perceived Value -> Purchase Intention	0.028	0.966	0.334 ^ns^	Not supported
H_17_	Physical Environ -> Perceived Value -> Purchase Intention	0.003	0.074	0.941 ^ns^	Not supported
H_18_	**Perceived Product Quality -> Perceived Value -> Purchase Intention**	**0.252**	**4.670**	0.000***	**Supported**
H_19_	Customer Trust -> Perceived Value -> Purchase Intention	0.066	1.380	0.168 ^ns^	Not supported

Notes: ***p < 0.001; (^ns^): not significant.

## 2 Experimental Design, Materials and Methods

The dataset [8] presented is quantitative and collected through a self-administered questionnaire. The questionnaire consisted of sections, namely, sections A and B. Section A contained information about the demographic profile of respondents, including gender, marital status, age, level of education, employment status, type of customer, and shopping frequency. For the demographic profile of respondents, the Statistical Package for the Social Sciences (SPSS) was used to analyze the data. Section B included the measurement instruments used for the constructs (empathy, convenience, price sensitivity, physical environment, perceived product quality, customer trust, perceived value, and purchase intention). A non-probability convenience sampling technique was used to obtain data from consumers of grocery stores located in the largest township in South Africa, Soweto, in the city of Johannesburg [9]. Soweto is an acronym for South-Western Townships, and comprises about forty periurban townships [10]. There was no sampling frame to draw from; hence, a convenience approach was more suitable. To increase the response rate, the participants were approached in different settings (in and outside the grocery stores, the comfort of their homes, and the streets of Soweto). Therefore, the convenience sampling approach enabled the researchers to target specific respondents with crucial information and shopping experience in township based grocery stores to provide relevant feedback to enrich the data [11]. However, since the research applies a convenience sampling technique, the results can only be generalized to the subpopulation from which the sample was drawn [12]. The targeted sample size was initially 300, and only 281 data points were usable, indicating a response rate of 94%. Partial Least Squares Structural Equation Modellssing (PLS-SEM) can work with an extensive range of sample sizes efficiently, from small (*n*< 100) to large, indicating that 281 data points are adequate to perform PLS-SEM [13]. SmartPLS 3 software was used to analyze the data. A pilot test was conducted to ascertain the reliability and validity of the measurement instruments in preparation for the full-scale data collection.

Before assessing the measurement model, the common method variance (CMV) was evaluated. Harman's one-factor test was conducted to assess the possibility of common method bias. The results demonstrated that the total variance explained by a single factor was 42.24%, which is below the recommended threshold of 50% [14,15], implying that there are no issues of common method bias. The first step in PLS-SEM is assessing the reflective measurement model [16]. This includes evaluating the measurement model, which contains estimating indicator loadings (> 0.70) as shown in Fig. 2, which were all acceptable [16] as outlined in Table 3. The second phase is the composite reliability (CR) for internal consistency assessment, which should be (>0.70) and Cronbach's alpha (> 0.70) [17]. As shown in Table 3, all conditions were met. The third phase of the reflective measurement model assessment includes evaluating the convergent validity of constructs, which is tested using the average variance extracted (AVE), which should be (> 0.50) [14], as demonstrated in Table 3. All the AVE values were above 0.50 and met the conditions. The fourth phase of the reflective measurement model evaluation assesses the discriminant validity using the Fornell and Larcker (1981), Heterotrait-Monotrait (HTMT), and cross-loadings as indicated in Tables 4^5^, and 6, respectively. The discriminant validity for all the tests was confirmed [14], [16], [18].

To analyze the proposed hypotheses, the structural model was assessed. Before proceeding with the analysis, it is crucial to check multicollinearity. To check multicollinearity, the Variance Inflation Factor (VIF) values were evaluated, and all the values for VIF were (< 5) as outlined in Table 3, indicating that there are no issues of collinearity [14]. VIF values greater than or equal to 5 indicate critical collinearity issues [16]. The R^2^ and Q^2^ assessments for perceived value and purchase intention were (0.497; 0.324) (0.566; 0.519) and demonstrated a moderate and medium effect [16], as shown in Fig. 3. The Standardized Root Mean Square Residual (SRMR) was also acceptable at 0.077, which is below the recommended threshold of (< 0.080) [19]. To assess the significance of the hypothesized relationships, bootstrapping was used with a minimum sample of 5 000. The direct effects and mediation tests are presented in Tables 7 and 8.

## Ethics Statement

Informed participant consent was obtained. The participants were informed that participation was voluntary and could withdraw at any given point from the survey. Anonymity was also guaranteed as no personal identifiable information was requested. The School of Business Sciences ethics committee (Wits University) approved the ethics clearance certificate under protocol number (CBUSE/1270).

## CRediT authorship contribution statement

**Valencia Melissa Zulu:** Conceptualization, Methodology, Supervision, Investigation, Software, Formal analysis, Writing – review & editing. **Andriaan Mpho Nkuna:** Conceptualization, Methodology, Investigation, Data curation.

## Declaration of Competing Interest

The authors declare that they have no known competing financial interests or personal relationships, which have, or could be perceived to have, influenced the work reported in this article.

## Data Availability

Subsistence Retail Consumer Data (Original data) (Mendeley Data).
